# Mechanisms of Developmental Toxicity of Dioxins and Related Compounds

**DOI:** 10.3390/ijms20030617

**Published:** 2019-01-31

**Authors:** Wataru Yoshioka, Chiharu Tohyama

**Affiliations:** Laboratory of Environmental Health Sciences, Center for Disease Biology and Integrative Medicine, Graduate School of Medicine, The University of Tokyo, Tokyo 113-0033, Japan; tohyamac-tky@umin.org

**Keywords:** dioxin, TCDD, malformation, terata, cleft palate, hydronephrosis, prostate, heart, jaw

## Abstract

Dioxins and related compounds induce morphological abnormalities in developing animals in an aryl hydrocarbon receptor (AhR)-dependent manner. Here we review the studies in which 2,3,7,8-tetrachlorodibenzo-*p*-dioxin (TCDD) is used as a prototypical compound to elucidate the pathogenesis of morphological abnormalities. TCDD-induced cleft palate in fetal mice involves a delay in palatogenesis and dissociation of fused palate shelves. TCDD-induced hydronephrosis, once considered to be caused by the anatomical obstruction of the ureter, is now separated into TCDD-induced obstructive and non-obstructive hydronephrosis, which develops during fetal and neonatal periods, respectively. In the latter, a prostaglandin E_2_ synthesis pathway and urine concentration system are involved. TCDD-induced abnormal development of prostate involves agenesis of the ventral lobe. A suggested mechanism is that AhR activation in the urogenital sinus mesenchyme by TCDD modulates the wingless-type MMTV integration site family (WNT)/β-catenin signaling cascade to interfere with budding from urogenital sinus epithelium. TCDD exposure to zebrafish embryos induces loss of epicardium progenitor cells and heart malformation. AHR2-dependent downregulation of Sox9b expression in cardiomyocytes is a suggested underlying mechanism. TCDD-induced craniofacial malformation in zebrafish is considered to result from the AHR2-dependent reduction in SRY-box 9b (SOX9b), probably partly via the noncoding RNA *slincR*, resulting in the underdevelopment of chondrocytes and cartilage.

## 1. Introduction

Dioxins and related compounds are a group of structurally related chemicals composed of two coplanar benzene rings. These compounds induce a similar spectrum of toxicity phenotypes with a wide degree of potency. Each chemical is assigned a toxic equivalency factor (TEF) relative to 2,3,7,8-tetrachlorodibenzo-*p*-dioxin (TCDD), the most toxic compound among this group of chemicals [1]. In experimental studies on toxicity mechanisms, TCDD has been used as a prototypical compound, with teratogenicity representing a sensitive indicator of TCDD toxicity in experimental animals [2]. Aryl hydrocarbon receptor (AhR, or dioxin receptor), a ligand-activated transcription factor (see reviews [3,4] for molecular biology of AhR and history), is indispensable for the manifestation of TCDD teratogenicity. This pivotal role for AhR was demonstrated using AhR-null mice [5,6] and AHR2-null zebrafish [7]. The lack of teratogenicity in the absence of AhR indicates that intrinsic factors inadequately regulated by TCDD-activated AhR are involved in the pathogenesis. Downstream of AhR signaling, putative candidate molecules have been indicated to distinctly induce various toxicity phenotypes [8]. The present review focuses on the recent progress reported on the mechanisms of developmental toxicity, in terms of teratogenicity, malformation, and morphological changes in laboratory animals exposed to TCDD.

## 2. Cleft Palate

### 2.1. Characterization of TCDD-Induced Cleft Palate

Cleft lip and cleft palate are among the most common birth defects in humans [9,10]. Pathogenesis of isolated cleft palate (cleft palate only without cleft lip) and cleft lip accompanied with or without cleft palate are thought to result from different mechanisms based on observations that these two conditions do not segregate in the same family. In hamsters, mice, and rats, TCDD administration to pregnant animals induces cleft palate in fetuses [11,12,13]. TCDD was confirmed to induce cleft palates but not cleft lips in mice [14,15].

The susceptibility peak period for the onset of cleft palate is around gestational days (GDs) 9–12 in ICR and C57BL/6 strains of mice [14,15,16,17]. In a report by Couture et al. [16], oral administration of TCDD at a dose of 24 μg/kg to pregnant C57BL/6 mice on GDs 8 and 12 results in 93% and 100% incidence, respectively, when the fetuses were examined on GD 18. In contrast, TCDD administration on GDs 6 and 14 results in 40% and 0% incidence, respectively. The incidence in the earlier period is low to modest (0–40%) at a TCDD dose of up to 40 μg/kg [14,16,17], whereas it is high (100%) at a dose of 80 μg/kg [17], suggesting that the higher dose cancels the apparent window of susceptibility in the earlier period. The lower or no incidence in later periods irrespective of the lower or higher dose [14,15,16,17] is considered to be related to the timing of palatogenesis completion [18].

Palatogenesis is considered to comprise several steps: growth, shelf elevation, contact, and fusion [19] (see review [20] for detailed mechanisms). The timing of organogenesis has been studied in detail in mouse fetuses [21]: in the normal development, palate shelves elevate from GD 14.0 to GD 14.25 and fuse completely by GD 14.5 [15,21]. TCDD exposure delays the elevation of shelves by one day, reduces the fusion efficiency of shelves on GD 14–16, and induces cleft palate on GD 17–18 [21,22,23,24]. Of note, even successfully fused palate shelves are suggested to be finally dissociated to develop cleft palate in TCDD-exposed fetuses [15,22,23]; these fusion rates in fetuses exposed to TCDD at a dose of 40 μg/kg were 17.5% and 0% on GD15 and GD18, respectively [22]. These findings suggest that TCDD not only perturbs the palatogenesis steps but also induces post-fusional rupture of the palate [25]. In addition, mouse embryos with narrower skulls owing to exencephaly are resistant to TCDD-induced cleft palate [24], which indicates the importance of balance in the development of the palatal shelves and the skull size.

### 2.2. Molecular Basis of TCDD-Induced Cleft Palate Onset

Genetic ablation of AhR results in the complete suppression of TCDD-induced cleft palate [5], demonstrating an indispensable role of AhR in the pathogenesis. AhR promoter activity is high in epithelial cells and weak in mesenchymal cells of palatal shelves [5]. Furthermore, antibody immunoreactivity against AhR is found in bone and muscle tissues in the palate [23]. Importantly, AhR in the medial edge epithelia (MEE) in palatal shelves are indicated to be more strongly expressed before rather than after the fusion of palatal shelves [5]. Furthermore, MEE in TCDD-exposed fetuses are covered with a monolayer of epithelial cells [21] with filopodia extensions reduced in number and length [21,26], whereas in the vehicle-control fetuses, they are covered with 2−3 layers of epithelial cells with filopodia extensions [21,26]. These findings are consistent with previous observations that TCDD inhibits proliferation of cells in palatal shelves [24] and with the interpretation that TCDD perturbs programmed cell death in MEE [27,28].

Thomae et al. [26] utilized ex vivo culture of palatal shelves harvested from mouse fetuses to show that TCDD-exposed palatal shelves did not fuse, but that control palatal shelves did. In addition, TCDD exposure ex vivo reduced filopodia extension in MEE similarly to that in vivo. On the other hand, the layer structure of epithelium exposed to TCDD ex vivo [26] appears to be thicker than that in vivo [21], and potentially important paracrine factors could be absent in the ex vivo culture. In the ex vivo experiments, the treatment of the palatal shelves with transforming growth factor-β3 (TGF-β3) restored palatal fusion in the presence of TCDD [26]. This finding and the central role of TGF-β3 in the fusion process of palatogenesis [20] suggest that insufficient amounts of TGF-β3 are involved in TCDD-induced cleft palate onset. However, because TGF-β3 expression is upregulated at mRNA [29,30] and protein levels [30] in the palate of TCDD-exposed fetuses, this hypothesis does not seem to be valid. This inconsistency might be explained by the differences in the ex vivo and in vivo experimental systems.

Furthermore, several genes and molecules are suggested to be associated with TCDD-induced cleft palate. A plausible candidate, fibroblast growth factor receptor 1 (FGFR1), is expressed in the frontal epithelia before shelf elevation, and FGFR1 mutations are often associated with cleft palate. Disappearance of FGFR1 was observed in TCDD-exposed shelves on GD 15.5 [21]. The expression of the osteogenesis markers Runx2 and osteopontin (OPN) is decreased in the palatal bone wherein AhR is strongly expressed on GD16 [23]. The expression of the myogenesis markers MyoD and desmin is diminished in TCDD-exposed palatal shelves on GD 16 [21]. Higher level in the expression of epidermal growth factor (EGFR) and the associated decrease in E-cadherin, α-catenin, and β-catenin expression in TCDD-exposed palatal shelves may be involved in the onset of TCDD-induced cleft palate [22,27]. Burns et al. [31] pointed out the possibility that reduction in the expression of SRY-box 9 (Sox9) may be involved in TCDD-induced cleft palate based on the similarities in development as well as gene expression patterns between the mouse palate and the zebrafish parasphenoid. Finally, although expression of a number of genes is reportedly altered in the palate exposed to TCDD, the toxicological significance of such alterations remains to be clarified.

## 3. Hydronephrosis

In humans, hydronephrosis is found in 1.5–3.3% of all autopsies [32] and in 1–5% of pregnancies on antenatal ultrasonography [33,34]. The major cause of hydronephrosis is the anatomical obstruction of the urinary tract from the kidney to the bladder via the ureter. Any obstruction of the urinary flow at any point along the urinary tract causing retention of urine will increase the retrograde hydrostatic pressure, leading to the destruction of kidney parenchyma and hydronephrosis. In mice, more than 100 genes are associated with hydronephrosis [35,36], and a mutation in or lack of any one of these genes results in hydronephrosis development. They encode a wide variety of proteins, including components of the renin–angiotensin system, ligands and receptors in signaling cascades, structural proteins of the ureter, extracellular matrix proteins, and transporters. Such a variety suggests that the urinary tract requires multiple mechanisms to perform urine propelling and to maintain an adequate balance of urine production and excretion and that the loss of any one of these mechanisms results in the development of hydronephrosis. One of these mechanisms is related to polyuria. A mutation in or lack of genes, such as those for aquaporin 2 (AQP2), Barttin, sodium-potassium-chloride cotransporter 2 (NKCC2), or V2 vasopressin receptor, results in polyuria and hydronephrosis [37,38,39,40,41,42], and polyuria can overwhelm the urine excretion capacity to induce hydronephrosis. Herein, we review studies on the toxicological basis of the TCDD-induced hydronephrosis in mice and rats.

### 3.1. TCDD-Induced Hydronephrosis

Courtney and Moore [13] found that administration of TCDD to pregnant mice or rats induces kidney abnormalities including hydronephrosis in fetuses. Moore et al. [43] further characterized TCDD-induced hydronephrosis. The oral administration of TCDD at doses of 1–3 μg/kg to pregnant C57BL/6 mice on GD 10 or from GD 10 to GD 13 induces a size reduction or the loss of the renal papilla and the dilation of renal calyces and renal pelvis in the fetus on GD 18. In addition, oral dosing of TCDD of 1, 3, or 10 μg/kg to mothers at parturition induces hydronephrosis in their pups on postnatal day (PND) 14, revealing that TCDD exposure in the neonatal period induces hydronephrosis. In a four-way cross fostering study that comprises four groups of mother/pup pairs formed at the time of parturition [(1) non-exposed mother/non-exposed pup, (2) non-exposed mother/exposed pup, (3) exposed mother/non-exposed pup, and (4) exposed mother/exposed pup], it was demonstrated that pups are more susceptible to TCDD-induced hydronephrosis in the neonatal period than in the fetal period [43]. This observation in mice was extended to Holtzman rats [44]. These studies clarified that TCDD induces hydronephrosis in fetal and neonatal periods of rodents and that the latter is more sensitive than the former.

### 3.2. Characterization of TCDD-Induced Neonatal Hydronephrosis

Couture-Haws et al. [45] determined a critical window for TCDD-induced neonatal hydronephrosis (TiNH). When mothers of C57BL/6N mice were orally administered TCDD (9 μg/kg) on PND 1, their pups developed hydronephrosis by PND 26 with an incidence of 89%. When exposed on PND 4, the incidence dropped to 41%. When exposed on PND 8 or 14, the incidence was similar to that in vehicle-control mice. Thus, TiNH displays a narrow critical window confined to a few days after parturition.

TiNH in mice is not accompanied with anatomical obstruction; the histological examination of ureter serial sections revealed no obstruction from renal pelvis to bladder in the afflicted pups [6]. Lack of obstruction at the renal pelvis, the proximal portion of the ureter, and the ureter insertion site into the bladder is reported in other studies [43,46]. Furthermore, ink-injection into the pelvicalyceal space resulted in dyeing of the bladder at similar hydrostatic pressure in affected pups and vehicle-control pups [6]. The microscopic observation of ureteric peristalsis, which propels urine from kidney to bladder, revealed no apparent abnormality in the affected pups [46]. While these studies did not find any obstruction, TiNH was found to be accompanied with an increase in urine volume [46]. TCDD-exposed pups had greater volumes of urine in their bladder both at daytime and at night. Suppression of urine production by an antidiuretic agent resulted in reduction of the urine volume as well as of the incidence and severity of hydronephrosis. These studies revealed that TiNH is of a non-obstructive type and is associated with an increase in urine volume.

### 3.3. Molecular Targets Linking TCDD Exposure and TiNH

AhR is required for the onset of TiNH [6]. Additional factors responsible for TiNH are cyclooxygenase-2 (COX-2, coded by *Ptges2*) and microsomal prostaglandin synthase-1 (mPGES-1, coded by *Ptges*). COX-2 converts arachidonic acid (AA) to prostaglandin H_2_ (PGH_2_), and mPGES-1 transforms PGH_2_ to prostaglandin E_2_ (PGE_2_). TCDD exposure increases the expression of COX-2 and mPGES-1 in the kidney and the excretion of PGE_2_ from the kidney [6,47]. Pharmacological inhibition of COX-2 [6] or genetic ablation of mPGES-1 [47] suppresses the TCDD-induced increase of PGE_2_ excretion and completely blocks TiNH onset. Therefore, the COX-2/mPGES-1/PGE_2_ pathway plays an essential role in the onset of TiNH. An upstream regulator acting as the predominant regulator of this pathway in TiNH is cytosolic phospholipase A_2_α (cPLA_2_α, coded by *Pla2g4a*), which converts phospholipids into AA. The genetic ablation of cPLA_2_α results in marked suppression of TCDD-induced increase in the expression of COX-2 and mPGES-1 in the kidney and excretion of PGE_2_, and subsequently in hydronephrosis onset [48]. In vitro studies support the role of cPLA_2_α: TCDD-induced increase in COX-2 expression is suppressed by pharmacological inhibition or siRNA-mediated knockdown of cPLA_2_α, and AA plays a role in increasing COX-2 expression [49,50]. Furthermore, cPLA_2_α is transcriptionally [51,52] and/or enzymatically [49,50] upregulated by TCDD via AhR. Of note, TCDD is suggested to impair milk production in dams [53] owing to defects in the mammary glands [53,54,55]. This raises the possibility that TCDD-induced impairment in lactation influences TiNH. However, the potential influence seems to be too small to be detected in pups lacking one of essential factors such as AhR and mPGES-1 [6,47].

Interestingly, while TCDD-induced upregulation of the COX-2/mPGES-1/PGE_2_ pathway depends on cPLA_2_α, TCDD-induced increase in the expression of other genes, such as those coding for cytochrome P450 1A1 (CYP1A1), AhR repressor (AhRR), and insulin-like growth factor binding protein 1 (IGFBP-1), does not depend on cPLA_2_α in the pup kidneys [48]. These cPLA_2_α-independent genes are known as direct targets of transactivation capacity of AhR [56,57]. Detailed mechanism(s) for the cPLA_2_α-dependent pathway are not yet elucidated, but several possibilities are conceivable. cPLA_2_α may be directly upregulated by transactivation capacity of AhR. Alternatively, cPLA_2_α may be activated by noncanonical actions of AhR, such as “nongenomic action of AhR”, in which ligand-bound AhR rapidly induces calcium signaling in the cytoplasm to induce activation of cPLA_2_α [58].

Signaling downstream of PGE_2_ leading to TiNH remains to be elucidated. A plausible mechanism is that increased PGE_2_ in the renal tubules interferes with the water reabsorption system [59,60,61], resulting in a decrease in water permeability [60,61] to produce diluted urine [46,62]; consequently, the increased urine volume [46] overwhelms the ureter propelling capacity and causes backpressure in the kidney pelvicalyceal space.

### 3.4. Molecular Basis of the TiNH Window and TiNH Susceptibility

Assessing developmental stage-specific onset of toxicity phenotypes is challenging but essential in the risk assessment of TCDD. Based on the understanding of the molecular mechanism of TiNH, neonatal stage-specific factors were researched [46]. In adult mice, which are resistant to TCDD-induced hydronephrosis [45], TCDD does not induce upregulation of COX-2 or mPGES-1 expression, neither does it increase urine volume. However, TCDD induces renal expression of AhR target genes [46], indicating that neonatal stage-specific upregulation of PGE_2_ synthesis is responsible for TiNH mediated by polyuria.

Remarkable differences in susceptibility among animal species and genetic backgrounds represent another challenge of TCDD toxicity evaluation. The lethal dose 50 (LD50) of TCDD for guinea pigs is 0.6 μg/kg [12,63], whereas that for hamsters is 1160–5050 µg/kg [64,65]. Even within species, sensitivity in response to TCDD can be considerably different: LD50 value for DBA/2 mice is 536 µg/kg and that for C57BL/6 mice is 114 µg/kg [66,67,68]. The susceptibility of humans to TCDD is predicted to be less than that of mice; this can be stated based on the case of Victor Yushchenko, who was intoxicated by TCDD with an estimated dose of about 25 μg/kg [69], and an experiment that utilized mice carrying humanized AhR [70]. Affinity of AhR to TCDD represents a determination factor for the sensitivity differences [67,71]. Little is known about additional factors determining sensitivity to TCDD toxicity. C57BL/6J and BALB/cA mice exhibit a substantial difference in the sensitivity to TiNH, although both strains possess AhRs with high affinity to TCDD [62]. This difference is attributed to the lower responsiveness of mPGES-1 expression to TCDD exposure in the kidney of BALB/cA pups. This finding proves the notion that factors responsible for a toxicity phenotype determine the susceptibility to toxicants [62].

### 3.5. Pathophysiology and Mechanisms of TCDD-Induced Fetal Hydronephrosis

A distinct characteristic of TCDD-induced fetal hydronephrosis (TiFH) is the presence of dilated ureters or hydroureter [43,72,73,74], which is not the case for TiNH at all [6,43,46]. In addition, Abbott et al. [72] found hyperplasia of ureter epithelium and occlusion of ureteric lumens in TCDD-exposed fetuses. The ureteric occlusion was further confirmed by ink injection from the bladder. Therefore, TiFH is an obstructive hydronephrosis with the following etiology. Ureter lumens are anatomically obstructed by TCDD-induced hyperplasia and the urine backpressure expands the ureter and pyelocaliceal space of the kidney, leading to renal parenchyma destruction.

In search for endogenous factors responsible for TiFH, AhR was identified as a molecule that is essential for TiFH development [5,75]. Although the mRNA level of CYP1A1 in the kidney of TCDD-exposed fetus is about 1,000 times higher than that in vehicle-control mice, CYP1A1 is not involved in TiFH onset because lack of CYP1 genes does not suppress TiFH onset [76]. As TCDD induces epithelial hyperplasia in the ureter in TiFH, growth factors have been investigated. TCDD increased the antibody immunoreactivity against EGFR [77] and increased the expression of EGFR ligands amphiregulin and epiregulin in fetus ureters [78]. However, in vivo experiments using mice lacking EGFR [79] and mice lacking epidermal growth factor (EGF) and/or fibroblast growth factor-α (FGF-α) [80] do not support the hypothesis of EGFR being required for TiFH. Additionally, cPLA_2_α does not play a causative role in TiFH because genetic ablation of cPLA_2_α has little influence on TiFH onset [74]. Furthermore, the expression level of mPGES-1 mRNA is not affected by absence of cPLA_2_α and/or by TCDD-exposure in the fetal kidney [74]. Thus, endogenous molecules, other than AhR, responsible for TiFH onset remain to be identified.

### 3.6. Hydronephrosis in Rats

TiFH and TiNH are also induced in rats [13,44]. Nishimura et al. [44] found that TCDD concentrations in the pups lactationally exposed to TCDD were similar in the cortex, outer zone of the medulla, and inner zone of the medulla of the kidney, whereas antibody immunoreactivity against CYP1A1, a typical AhR-dependent gene, was predominantly detected in the outer zone of the medulla. Based on the significant role of AhR in determining susceptibility to TCDD toxicities, activation of AhR in the outer zone of medulla may be responsible for the onset of hydronephrosis. It should be noted that AhR is required for the urinary tract development in a species-specific manner because the absence of AhR in rats causes hydroureter and hydronephrosis even in the absence of an exogenous ligand [81]. Such abnormalities in the urinary tract are observed in rats lacking AhR at 1, 6, and 12 weeks of age with 100% incidence. The etiological factors may be endogenous ligand(s) of AhR and/or protein(s) interacting with AhR. In contrast to rats, mice lacking AhR do not develop hydroureter or hydronephrosis in the absence of TCDD [5,6], suggesting that AhR has diverse roles in tissue development across species.

## 4. Abnormal Development of Prostate

In the process of prostate organogenesis, numerous prostate buds (prostate duct progenitors) develop from fetal urogenital sinus epithelium (UGE). The prostate buds initiate outgrowth into the surrounding urogenital sinus mesenchyme (UGM) and branching morphogenesis. In rodents, the prostate develops to form ventral, dorsolateral (dorsal and lateral), and anterior lobes (here, only referred to as ventral prostate and so on; see review [82] for detailed mechanisms of prostate organogenesis in human and mouse).

### 4.1. Characteristics of TCDD-Induced Abnormality of Prostate Development

*In utero* and lactational TCDD exposure of C57BL/6J mice at a maternal dose of 5 μg/kg on GD 13 results in a barely detectable ventral prostate in the male offspring on PNDs 35, 90, 100, and 510 [83,84], smaller weights of dorsolateral and anterior prostate on PND 35, fewer numbers of main ducts and ductal tips in lateral and dorsal prostate on PND 90, and consistently smaller overall structure of dorsolateral and anterior prostate on PND 90 [83]. A lower dose (1 μg/kg) of TCDD results in a decreased weight only in ventral prostate in adulthood, which might be associated with a decreased voiding pressure in the bladder [85]. A four-way cross fostering experiment indicated that ventral and anterior prostates are more sensitive to TCDD actions in the fetal period than in the neonatal period, whereas dorsolateral prostate is equally sensitive in these periods [86]. A more detailed study [87] revealed that TCDD exposure starting on GD 15.5 causes ventral prostate agenesis and that on GD 16.5 decreases ventral prostate weight by 50%. Dorsolateral bud formation is most responsive to TCDD between GDs 14.5 and 15.5, and exposure during the sensitive period causes displaced buds and decreased bud number. Taken together, the critical window for TCDD toxicity varies between prostate lobes during prostate development in mice.

### 4.2. Molecular Basis of TCDD-Induced Prostate Malformations

The following findings revealed that TCDD directly activates AhR in UGM to modulate paracrine signals, which inhibits prostatic bud formation in UGE. First, AhR-null mice are resistant to TCDD-induced abnormalities of prostate development (decrease in weight of prostatic lobes and altered expression levels of differentiation marker genes) [88]. Second, mRNAs of AhR and indicator genes of AhR activation exhibit histologically overlapping distributions in the periprostatic mesenchyme, which closely contacts UGE in the TCDD-exposed fetus [87]. Third, TCDD prevents prostatic epithelial buds from forming in cultured urogenital sinus derived from wild-type mice but not from AhR-null mice [89]. Fourth, TCDD-exposed organ cultures of dorsolateral or ventral UGM in combination with UGE result in loss of budding from the epithelium when the ventral mesenchyme is from wild type mice but not from AhR-null mice [90]. The loss of budding is observed regardless of the genotype of the epithelium, indicating that AhR expression not in the epithelium but in UGM has a role in the budding [90]. It should be noted that AhR has a role in prostate development, because loss of functional AhR causes delays in growth of prostatic lobes and seminal vesicle in the absence of an exogenous ligand [88]. Therefore, it is thought that TCDD acts on AhR to induce responses in the developing prostate in a complicated manner.

The Wnt/β-catenin signaling cascade may link AhR activation and ventral prostate agenesis. TCDD alters expression levels of genes regulating Wnt signaling around the timing and location of the budding [91]. In addition, treatment with an anti-Wnt5a antibody restores a TCDD-induced decrease in prostatic buds in an organ culture system [92]. These results suggest a possible mechanism involving Wnt signaling. β-catenin activation is indicated in the ventral basal epithelium immediately prior to the initiation of ventral prostatic budding, which is abolished by TCDD exposure [91]. Based on these findings, it is proposed that AhR activation alters Wnt ligands expression in UGM, which disrupts regulation of β-catenin signaling via the receptors for Wnt ligands in UGE and subsequently inhibits the budding [93].

## 5. Heart and Craniofacial Malformations

Fish species are extremely susceptible to TCDD exposure and exhibit similar toxicity phenotypes to those observed in other vertebrates, including wasting syndrome, delayed mortality, cardiovascular dysfunction, craniofacial malformations, and liver damage (see the review by King-Heiden et al. [94]). AHR2 in fish is the functional orthologue of mammalian AhR [95]. This section will focus on the heart and craniofacial malformations that have been extensively investigated in zebrafish (*Danio rerio*) embryos. TCDD exposure levels in this section are expressed as TCDD concentrations in water based on the studies cited in this review although the actual TCDD concentrations in water are uncertain owing to TCDD’s very high log Kow [96], while describing the exposure level as tissue TCDD concentration may be more applicable to risk assessment in the real world [97].

### 5.1. Heart Malformation

When fertilized zebrafish eggs are exposed for 1 h to waterborne TCDD (1 ppb, or 1 ng/g, nearly equivalent to 1 ng/mL) [98] shortly after fertilization, severe heart malformation is observed in the embryos. Exposure at 7, 12, and 30 days post-fertilization results in less severe, far more modest, and no detectable defects, respectively [98]. Major characteristics of the heart malformation at 72 h and 96 h post-fertilization (hpf) in the embryo exposed to 1 ppb TCDD for 1 h involve a failure to form the looping shape of the heart, elongated atrium, and shrunken ventricle [99]. TCDD exposure reduces the number of cardiac myocytes based on the marker gene *cmlc2* expression at 48 hpf, which precedes observable effects on peripheral blood flow by one day [99]. The cells primarily affected by TCDD are those of the proepicardium [100]. TCDD exposure at 24 hpf (before the proepicardium formation) almost diminishes proepicardium on the ventricle or atrium at 50 hpf or 72 hpf, whereas proepicardium is formed in control embryos at these times. Delaying the start of TCDD exposure from the 24 hpf to 48 hpf, 72 hpf, 96 hpf, and 122 hpf reduces this effect. The proepicardium cells on the myocardium, formed in the developing heart, subsequently form the epicardium. This process is lost when exposed to TCDD during proepicardium formation but not after epicardium formation, accounting for the windows of sensitivity to the severest form of heart malformation [98]. Thus, TCDD interferes with a distinct type of progenitor cells during development, which propagates to cause heart malformation. The major characteristics of heart malformation and loss of epicardium are not secondary to pericardial edema because suppression of the edema by osmotic support with mannitol does not alleviate these abnormalities [99,100]. TCDD exposure at a much lower dose (1 ng/L) for 4 days induces a reduction in ventricular length at 10 days post-fertilization [101], indicating that heart morphogenesis is extremely sensitive to TCDD exposure.

A genetically modified zebrafish embryo, carrying a constitutively activated AhR (caAHR) under the control of the cardiomyocyte-specific gene *cmlc2* promoter [102], fails to form epicardium and develops heart malformation characterized by an unlooped heart with an elongated atrium and a shrunken ventricle. Additionally, heart functions in the caAHR-zebrafish are inhibited similarly to those of TCDD-exposed wild-type zebrafish. A significant role of AHR2 in TCDD-induced heart malformation has been demonstrated using an antisense morpholino [95]. Recently, Souder and Gorelick [103] reported that a newly generated AHR2 mutant is resistant to 10 ng/mL TCDD exposure and does not produce abnormal heart looping or severe pericardial edema in contrast to the corresponding wild-type embryos and mutants of AHR1A and AHR1B. It is not clear whether the lack of functional AHR2 in other AHR2 mutant lines described above [7,104] also blocks heart malformation. The gene *Sry-box containing 9b* (*sox9b*) links AhR activation with heart malformation [105]; *sox9b* expression in the heart ventricle at 72 hpf is significantly reduced by TCDD exposure, and the *sox9b-null* mutant zebrafish shows heart malformation, such as failure to form the looping shape of the heart, elongated atrium, and shrunk ventricle. In addition, loss of *sox9b* prevents formation of proepicardium and epicardium [105]. Cardiomyocyte-specific Sox9b inhibition by a dominant-negative mutant protein [106] results in abnormalities similar to those of TCDD-induced heart malformation, indicating the pivotal role of sox9b in epicardium formation and subsequent heart development.

### 5.2. Craniofacial Malformation

When fertilized zebrafish eggs are exposed to waterborne TCDD (0.3−7.4 ng/g water), craniofacial malformations, such as foreshortened snout and underdeveloped lower jaw, are observed at 60–240 hpf [107,108,109]. The effective dose 50 (ED50) for craniofacial malformations of embryos exposed to TCDD from 24 hpf to 48 hpf is 1.9 ng TCDD/g embryo at 240 hpf [108]. No specific window for the underdevelopment of the lower jaw was identified within 0–96 hpf [107,108,109]. Detailed analyses of the jaw morphology revealed that cartilage had abnormal structures with a smaller size in the TCDD-exposed embryos [7,31,107,109]. Chondrocytes are the affected cell type in the cartilage, as their number and length are decreased by TCDD exposure [31]. In addition, endodermal perichondrial cells that cover chondrocytes are affected by TCDD due to which they develop a reduced proliferation ability [31]. Furthermore, TCDD reduces ossification of craniofacial osteoblasts [31], which is consistent with TCDD-induced ossification failure in another fish model, medaka (*Oryzias latipes*) [110]. These findings indicate that chondrocytes and the peripheral cells are sensitive to TCDD insult, leading to malformations. It should be noted that dimethyl sulfoxide (DMSO), a solvent used to dilute TCDD, has recently been shown to alter cartilage structures; Meckel’s and platoquadrate cartilages in DMSO-exposed zebrafish manifest slightly but statistically significant differences in their relative position compared to those in non-exposed zebrafish [7]. Possible interactions of TCDD and DMSO may complicate the interpretations of the experimental results.

It has been suggested that reduced blood flow plays a role in TCDD-induced jaw malformation. This suggestion is reasonable because cardiomyocyte-specific activation of AhR induces jaw underdevelopment in addition to the expected cardiac defects [102]. A zebrafish model of cardiac dysfunction develops abnormal jaw growth in the absence of TCDD [111]. On the other hand, the abnormality of the lower jaw develops before the cardiovascular abnormalities are not evident [107,109]. Taken together, the direct action of TCDD on the lower jaw and the indirect action via the circulation failure could contribute to TCDD-induced jaw malformation.

AHR2 mediates TCDD-induced craniofacial malformations in zebrafish as AHR2-null zebrafish are resistant to the toxicity [7]. Additionally, the zebrafish mutant line AHR2^hu3335^, with a mutation in the transactivation domain [104], and zebrafish embryos injected with an antisense morpholino targeting AHR2 [112] are completely and partially resistant to development of craniofacial malformations, respectively. These studies clearly established that AHR2 is responsible for the TCDD-induced craniofacial malformation. Interestingly, AHR2-null zebrafish have cranial skeletal bone structure abnormality in adulthood even when TCDD is not administered, demonstrating endogenous functions of AHR2 in the development of skeletal bones. SOX9b is a critical chondrogenic transcription factor; deletion of sox9b results in jaw abnormalities of a considerably more severe degree compared with those induced by TCDD exposure [113]. AHR2-dependent reduction in SOX9b expression is thought to contribute to jaw malformation because of the following reasons. An antisense morpholino against *sox9b* mimics TCDD-induced jaw malformation and restoration of *sox9b* expression rescues the developing jaw from TCDD exposure [109]. TCDD induced-jaw malformation accompanies significant reduction in *sox9b* expression only in wild-type control allele but not in AHR2-null allele [7]. A reporter gene for *sox9b* promoter activity shows expression in perichondrial cells surrounding craniofacial cartilages [31]. In addition, TCDD-induced reduction in both *sox9b* expression and abnormality in jaw structure are also observed in medaka (*Oryzias latipes*) [110], suggesting a common role of *sox9b* among fish species. The extent to which reduction in *sox9b* expression affects TCDD-induced craniofacial malformations remains to be uncovered; the craniofacial malformations are observed at a TCDD dose (0.25 ng/mL) at which a reduction in *sox9b* mRNA level in the whole embryo is not evident [114]. The noncoding RNA *slincR* may link transactivation capacity of AHR2 with reduction in *sox9b* expression; the *slincR* promoter has cis-elements for AHR2-binding and TCDD upregulates *slincR* expression in an AHR2-dependent manner [7,115]. In addition, *slincR* transcripts are suggested to be enriched in a region of the genome corresponding to the 5′-untranslated region (UTR) of *sox9b* [114] and an antisense-morpholino-mediated suppression of *slincR* expression cancels the reduction of *sox9b* expression in the presence of TCDD [115]. The knock-down of *slincR* using an antisense morpholino altered the structures of jaw cartilages in TCDD-exposed zebrafish [114]. The exact roles and contribution of the *slincR/sox9b* pathway should be examined further since *slincR* and *sox9b* only partially overlap with respect to expression time and location [115].

## 6. Perspectives for Future Research

Toxicological research on dioxin and related compounds was initiated for risk assessment after episodic accidents of humans exposed to these chemicals in the general and occupational environments. These episodes include accidents in Seveso, Italy [116,117], food poisoning accidents named *Yusho* and *Yucheng* in Japan and Taiwan, respectively [118], and Victor Yuchenko’s incidence [69], which suggest that daily exposure to these chemicals even at low doses may induce abnormalities, in particular, when exposed during the most sensitive period of development. In the risk assessment of dioxin and related compounds, developmental toxicity data obtained from animals exposed in utero and via lactation have been utilized [119]. In this risk assessment, the concept of body burden that reflects actual dioxin concentration in the blood circulation was practically used to minimize the effects of route of administration between humans and experimental animals. However, there exists a non-negligible limitation in the extrapolation of TCDD toxicity data from animals to humans. In TCDD toxicity studies, the animal species and strains that are sensitive to respond to TCDD have been often used on the standpoint to protect human health. The tolerable daily intake (TDI) value and tolerable monthly intake value are 4 pg toxic equivalency (TEQ)/kg per day [119] and 70 pg TEQ/kg per month [120], respectively. Because AhR structure significantly determines a difference in susceptibility between animal species and between strains, the selection of a particular kind of species or strains would affect the TDI value. Although there is no ideal species or strains that is multipotent for risk assessment, production of humanized AhR knock-in mice would be an option as an experimental model. In a humanized AhR mouse model [70], in which C57BL/6 AhR is replaced with human AhR, the TCDD-exposed animals turned out to be less susceptible than C57BL/6 and DBA/2 in terms of the onset of cleft palate and hydronephrosis. It should be noted that once human AhR is introduced into the murine cells, cofactors that supposedly interact with AhR are of murine origin, which suggest that these cells behave differently from those of human origins. As described in the above section, the AhR response to TCDD seems to totally differ between mice and rats because AhR-null rats manifest hydronephrosis without TCDD. The molecular mechanisms of AhR and cofactors with AhR ligands need further studies. The other limitation is the difficulty of determining an exact critical period during the developmental period. When dams are exposed during gestation, TCDD will be transferred to fetuses and neonates via placenta and via lactation, respectively. Although cross-fostering experiments have been performed to determine the critical period, it is often difficult to estimate the TCDD concentration at a target site of an organ during development. The fact that organogenesis during ontogeny does not necessarily match between rodents and humans is another factor that complicates interpreting toxicity data of animal models during gestation.

## 7. Conclusions

Dioxin and related compounds induce various toxicities in an AhR-dependent manner. Identification of AhR as a dioxin receptor and the use of AhR-null animals and cells have clearly showed that AhR is essential to induce majority of the toxicities and achieved a breakthrough in the field of dioxin toxicology. The physiologic role of AhR remains unclear. On the other hand, it avidly binds TCDD to mediate signals downstream of AhR to induce abnormalities. The mechanisms of these abnormalities have been intensively unveiled for cleft palate, hydronephrosis, abnormal development of prostate, and heart and craniofacial malformation. In some cases, AhR is likely to function in the absence of dioxin-like chemicals. Elucidating the mechanisms of endogenous and naturally occurring AhR ligands and clarification of the AhR signaling network consisting of cross talks of endogenous and exogenous ligands will be the next challenges. 

Recent studies have progressed in elucidating the molecular basis of specific toxicity phenotypes by combining genetic strategies targeting genes potentially involved in the mechanisms and detailed investigations of the pathophysiology of the toxicity phenotypes and/or cell types affected. Entire pathways from TCDD-exposure to the onset of toxicity phenotypes are to be unraveled in the near future. Humans and wild life have been exposed to dioxins and related compounds at a very low concentration. Although extrapolation of data from animal models to humans requires scrutiny in future studies, the use of TCDD as a prototype of these chemicals will be helpful for studying the molecular mechanism of toxicities that might be common in animals and humans.

## Abbreviations

AhR	Aryl hydrocarbon receptor
GD	Gestational day
PND	Postnatal day
hpf	Hour(s) post-fertilization
